# Psychiatric adverse events linked to glucagon-like peptide 1 analogues: a disproportionality analysis in American, Canadian and Australian adverse event databases

**DOI:** 10.1007/s11096-025-01943-x

**Published:** 2025-06-16

**Authors:** Jonah Katranski, Sihua Liang, Deirdre Morris, Vijayaprakash Suppiah, Chiao Xin Lim

**Affiliations:** 1 Pharmacy, School of Health and Biomedical Sciences, RMIT University, Bundoora, VIC 3083 Australia; 2 Clinical and Health Sciences, University of South Australia, Adelaide, SA 5001 Australia; 3 Australian Centre for Precision Health, University of South Australia, Adelaide, SA 5001 Australia; 4 Medicine Department, School of Clinical Sciences, Monash University, Clayton, VIC 3168 Australia

**Keywords:** CVAROD, DAEN, FAERS, GLP-1 analogues, Pharmacovigilance, Psychiatric adverse events

## Abstract

**Background:**

Glucagon-like peptide 1 (GLP-1) analogues are a class of medications that stimulate glucose-dependent insulin release and slow gastric emptying. With the increasing use of GLP-1 analogues, concerns about potential psychiatric adverse events (AEs) remain under-explored.

**Aim:**

This pharmacovigilance study aimed to investigate the prevalence of psychiatric AEs associated with currently available GLP-1 analogues by analysing publicly available national datasets from the US (FAERS), Canada (CVAROD) and Australia (DAEN).

**Method:**

Psychiatric AE reports were extracted from all three databases for all approved GLP-1 analogues. A disproportionality analysis was conducted to calculate reporting odds ratios (RORs) and 95% confidence intervals (CIs) for psychiatric AEs of interest.

**Results:**

Significant associations were identified when multiple databases reported elevated RORs. Semaglutide was associated with depressive symptoms (FAERS, ROR = 6.24 CI 4.49–8.69), panic attacks (FAERS, ROR = 1.46 CI 1.16–1.82) and suicidal ideation (FAERS, ROR = 2.58 CI 2.31–2.88). Liraglutide was linked to depression (CVAROD, ROR = 1.68 CI 1.12–2.51), while dulaglutide showed positive associations with eating disorders (FAERS, ROR = 1.47 CI 1.26–1.71) and insomnia (FAERS, ROR = 2.93 CI 2.35–3.66).

**Conclusion:**

GLP-1 analogues, particularly semaglutide and liraglutide, are associated with significant psychiatric AEs, especially depression and suicidal ideation. Further studies are required to understand the mechanisms underlying these associations, particularly in patients with pre-existing psychiatric conditions.

**Supplementary Information:**

The online version contains supplementary material available at 10.1007/s11096-025-01943-x.

## Impact statements

- By demonstrating potential associations between several psychiatric adverse events and glucagon-like peptide 1 analogues, this research supports the need for further investigations to establish these associations.
- It is important to consider the patient’s psychiatric health and history before prescribing to ensure the prescribing of glucagon-like peptide 1 analogues is appropriate.
- This study compares the risks associated with each member of the class, highlighting the potential differences in psychiatric adverse event profiles of various glucagon-like peptide analogues, and suggests that further prospective studies are warranted for confirmation.

## Introduction

The class of drugs known as glucagon-like peptide 1 receptor agonists (GLP-1 RAs), also called GLP-1 analogues, are synthetic analogues of GLP-1 which act by stimulating glucose-dependent insulin release from the pancreas and slowing gastric emptying [1]. The primary aim of these medications is to increase glucose-dependent insulin release and to inhibit inappropriate glucagon secretion to achieve ideal diabetic control [1]. Alongside glycaemic control, GLP-1 analogues have also been shown to have a favorable cardiovascular profile and contribute to weight loss [1, 2]. As a result, prescribing of these medications has increased exponentially in recent years. In the US alone, monitoring has found that GLP-1 analogue use in people with type 2 diabetes and atherosclerotic cardiovascular disease nearly doubled, rising from 5.2% in 2018 to 9.9% in 2022 [3]. Moreover, posts on social media have triggered a significant ‘off-label’ demand for GLP-1 analogues to achieve weight loss, further increasing the number of GLP-1 analogue users [4].

GLP-1 analogues have had extensive investigations into their safety and efficacy with several diverse adverse events (AEs) being associated with their use. Gastrointestinal AEs are by far the most common, ranging from nausea and diarrhoea (in up to 50% of users) to abdominal pain and constipation (in up to 10% of users) [5, 6]. Additionally, there have been reports of rare AEs such as pancreatitis and cancer that were thought to be associated with GLP-1 analogues [6, 7]. However, the lack of investigation into potential psychiatric AEs raises a concern for those with pre-existing psychiatric conditions that could benefit from GLP-1 analogue use. Even though some recent studies have explored the psychiatric effects of these medications, these studies have been contradictory and inconclusive [8–10]. Two studies have reported the beneficial effects of GLP-1 analogues on depression and anxiety in a corticosterone-induced depression mice model or in patients newly diagnosed with type 2 diabetes [8, 9]. Another study by Wang et al. has also found that GLP-1 analogues, specifically semaglutide, are linked to a significantly lower risk of suicidal behaviors compared to non-GLP-1 analogues for either treatment of obesity (e.g. naltrexone, bupropion, etc.) or diabetes (e.g. metformin, insulin etc.) [10]. Conversely, one study found an association between their use and increased psychiatric effects, including nervousness, insomnia, and eating disorders [11]. Additionally, a study performed by Ruggiero et al. [12] found that semaglutide and liraglutide were associated with a two-to-four-fold increase in the reporting probabilities of suicidal events compared to exenatide and dulaglutide. Similarly, a recent study by Guirguis et al. [13] showed a potential relationship between liraglutide, semaglutide, and tirzepatide and suicidal thoughts and behaviors, further highlighting the need for further research.

Analysing ‘real-world’ data may lead to the emergence of previously unreported AEs, sparking interest in further investigations for new user groups. The US Food and Drug Administration Adverse Events Reporting System (FAERS), Health Canada’s Vigilance Adverse Reaction Online Database (CVAROD) and Australian Therapeutic Goods Administration’s Database of Adverse Event Notifications (DAEN) are government databases to which healthcare professionals can report suspected post-market AEs with the aim of helping agencies and researchers identify safety concerns in pharmaceutical products [14–16]. Many pharmacovigilance studies have previously used data available in these government databases to report AE trends in a ‘real-world’ setting [17, 18].

A recent pharmacovigilance study examining psychiatric AEs associated with semaglutide, liraglutide and tirzepatide in the European Medicines Agency EudraVigilance database [19] reported depression, anxiety and suicidal ideation as the most common psychiatric AEs, with 20 out of 372 reports had a fatal or life-threatening outcome. However, as a disproportionality analysis was not completed, it was not possible to determine if there is an association between the use of these GLP-1 analogues and the psychiatric AEs examined. Further, this study only explored psychiatric AEs in three GLP-1 analogues, rather than all six agents within the class. Another pharmacovigilance study [11] investigated psychiatric AEs associated with GLP-1 analogues using the FAERS up until Q1 2023 found associations between these drugs and eight categories of psychiatric AEs. Given the rapidly increase in GLP-1 analogue usage globally, examination of psychiatric AEs in multiple large spontaneous adverse events reporting databases is required to gain a current understanding of the relationship between the use of GLP-1 analogues and psychiatric AEs.

### Aim

Combining and comparing AE reports from multiple databases enriches the analyses by increasing the sample size, breadth of data representation, and statistical power to detect associations. Hence, this study aimed to investigate the prevalence of psychiatric AEs associated with currently available GLP-1 analogues by analysing publicly available national datasets from the US (FAERS), Canada (CVAROD) and Australia (DAEN).

### Ethics approval

Ethical approval was not required for our study as it did not involve human participants, animals or the collection of personal data. Exemption was granted by the RMIT University ethics committee.

## Method

### Data sources

AE reports for all approved GLP-1 analogues (exenatide, semaglutide, liraglutide, lixisenatide, dulaglutide and tirzepatide) were downloaded from three online databases: FAERS, CVAROD and DAEN. The study period for each drug consisted of data from the launch date of each drug in the respective countries to the latest available data at the time of the search (Supplementary Table 1).

### Search strategies

A search of all psychiatric AEs was conducted for the GLP-1 analogues in the respective databases. To expedite and ensure uniformity across all databases, the Medical Dictionary for Regulatory Activities (MedDRA) classification of psychiatric events was used since all three databases had used the MedDRA classification system to categorise the reported AEs. Any events that did not fit into this classification were excluded. Similar AEs were clustered together in categories for ease of understanding (e.g. insomnia include insomnia, initial insomnia, middle insomnia and terminal insomnia).

The number of psychiatric events were recorded alongside the number of patients experiencing these events, their age and sex. Since concomitant medications and patient medical history were not fully known or recorded, these patient factors were not included in the analyses.

### Data analysis

A disproportionality analysis model was employed to identify associations between GLP-1 analogue use, and the occurrence of a specific AE compared to all other drugs (reported in that database). To ensure that associations are not driven by isolated findings from a single medication, all AEs from the three databases were reviewed and reported if they met all of the following criteria:

1. At least 10 reports for a single medication in a single database, and
2. Had been reported for at least two medications in at least two databases.

The minimum total number of reports was typically defined as 3 for ROR analysis [11]. As this is a comprehensive analysis involving all GLP-1 analogues utilising data from three national databases, we have established this inclusion criteria to minimise the chance of false positive signals caused by small reporting numbers, and to provide a clearer overview of adverse events with potential clinical relevance.

Reporting Odds Ratio (ROR) with 95% confidence intervals (CI) were calculated for each AE of interest, for each medication across the three databases [20]. An ROR greater than 1 indicates a greater correlation between the drug and AE. The Reporting of A Disproportionality analysis for drug Safety signal detection using individual case safety reports in Pharmacovigilance (READUS-PV) checklist was used to report this disproportionality analysis study (Supplementary Data File 2) [21, 22].

The formula for calculating ROR is: $\frac{\left(\frac{a}{c}\right)}{\left(\frac{b}{d}\right)}$

 where *a* is the number of reported cases of a symptom associated with the medication of interest; *b* is the number of other symptoms reported for that same medication; *c* is the number of reported cases of the same symptom associated with all other medications, and *d* is the number of all other symptoms associated with all other medications. A resulting odds ratio of greater than 1 is considered to suggest that the medication in question is more likely to be associated with reports of that symptom than are other medications [20].

The 95% CI is calculated using the formula: ${e}^{\text{ln}\left(ROR\right)\pm 1.96\sqrt{\frac{1}{a}+\frac{1}{b}+\frac{1}{c}+\frac{1}{d}}}$

This formula gives us two values representing the upper and lower confidence interval. If the range between these values does not include 1 then our result is considered significant.

The US database is the largest dataset in this study, providing a larger sample size for calculating RORs leading to more robust results. In contrast, the Australian and Canadian datasets are significantly smaller, which limited the power of their individual findings. A small number of reported cases could return very high RORs but with a very wide range in the CI, signifying less reliable results. Because of this, the US RORs were used to guide targeted searches in the smaller datasets.

While findings in smaller datasets should be interpreted cautiously, alignment with the larger US dataset increased confidence in the observed associations. Any cross-dataset agreement suggests the findings may be applicable across different population groups.

## Results

### Basic information on AE reports

A total of 16,678 psychiatric reports were identified for the GLP-1 analogues of interest across all three databases: exenatide (n = 5492, 32.9%), semaglutide (n = 4067,24.4%), dulaglutide (n = 3024, 18.1%), tirzepatide (n = 1447, 8.7%), liraglutide (n = 2552,15.3%) and lixisenatide (n = 96, 0.6%) (Supplementary Table 2). The three databases recorded the following number of reports each: FAERS (n = 16,090, 96.5%), CVAROD (n = 363, 2.2%) and DAEN (n = 225,1.3%) (Supplementary Table 2). Majority of the reports were in females (n = 7990, 64.2%) and the median age across the three databases was 60 years [interquartile range (IQR) = 54–68] (Supplementary Tables 3 and 4).

A total of 14,510 psychiatric AEs were selected for analysis across the three databases after undergoing the data analysis process (Table 1). The top five AE categories were mood disorders (n = 7,147, 49.3%), followed by sleep-related disorders (n = 3,052, 21.0%), confusion and cognitive changes (n = 1,745, 12.0%), suicidal thoughts and behaviors (n = 955, 6.6%) and agitation and restlessness (n = 940, 6.5%).

**Table 1:** Comparative table of selected psychiatric AEs listed for each GLP-1 analogue in the three databases

	Exenatide			Semaglutide			Dulaglutide			Tirzepatide^(5)^		Liraglutide			Lixisenatide^(5)^	
	FAERS	DAEN	CVAROD	FAERS	DAEN	CVAROD	FAERS	DAEN	CVAROD	FAERS	DAEN	FAERS	DAEN	CVAROD	FAERS	CVAROD
Agitation and restlessness	394	1	–	151	3	11	152	2	–	70	1	141	2	6	6	–
Agitation	90	**–**	**–**	28	1	1	33	1	**–**	15	**–**	33	2	1	2	**–**
Anger	48	1	**–**	22	**–**	3	22	1	**–**	12	**–**	21	**–**	1	**–**	**–**
Irritability	217	**–**	**–**	78	2	7	82	**–**	**–**	32	**–**	61	**–**	3	3	**–**
Restlessness	39	**–**	**–**	23	**–**	**–**	15	**–**	**–**	11	1	26	**–**	1	1	**–**
Mood disorders	2549	6	2	1554	37	76	1250	9	**–**	577	1	955	24	69	37	1
Mood altered	44	**–**	**–**	35	1	5	29	**–**	**–**	16	**–**	26	1	4	**–**	**–**
Mood Swings	39	**–**	**–**	26	1	1	24	1	**–**	8	**–**	36	**–**	2	**–**	**–**
Depressive disorders^(1)^	520	4	**–**	609	14	34	290	2	**–**	174	**–**	354	10	31	9	**–**
Apathy	13	**–**	**–**	26	1	2	12	**–**	**–**	5	**–**	14	1	3	**–**	**–**
Emotional disorder	23	**–**	**–**	18	2	**–**	17	1	**–**	6	**–**	13	**–**	1	**–**	**–**
Anxiety	574	1	1	434	11	19	327	2	**–**	217	1	291	9	21	8	**–**
Nervousness	695	**–**	**–**	113	**–**	3	163	1	**–**	33	**–**	90	**–**	2	10	**–**
Emotional distress	41	**–**	**–**	27	1	**–**	13	**–**	**–**	9	**–**	10	**–**	2	1	1
Panic attack	54	**–**	**–**	77	3	2	37	**–**	**–**	53	**–**	33	2	1	1	**–**
Post-traumatic stress disorder	8	–	–	6	1	–	17	1	–	2	–	8	1	–	–	–
Stress	462	1	1	157	2	8	282	–	–	45	–	70	–	2	6	–
Fear	76	–	–	26	–	2	39	1	–	9	–	10	–	–	2	–
Confusion and cognitive changes	744	3	–	262	11	13	378	6	1	89	–	214	5	8	11	–
Confusional state	424	2	–	141	4	6	194	3	–	37	–	115	–	4	6	–
Disorientation	183	–	–	33	–	1	34	–	–	14	–	32	–	2	–	–
Mental disorder	45	1	–	41	5	2	71	2	–	19	–	25	4	1	2	–
Delirium	18	–	–	12	1	1	10	1	–	4	–	15	–	1	1	–
Thinking abnormal	48	–	–	16	–	1	53	–	–	9	–	18	1	–	1	–
Abnormal behaviours	26	–	–	19	1	2	16	–	1	6	–	9	–	–	1	–
Eating disorder	83	1	–	44	–	5	172	1	–	36	–	33	–	2	2	–
Psychotic disorders	68	3	–	102	4	7	53	–	–	20	–	30	2	2	1	–
Hallucinations^(2)^	53	2	–	51	–	5	34	–	–	10	–	19	1	2	1	–
Paranoia	11	–	–	9	2	–	11	–	–	3	–	5	1	–	–	–
Psychotic disorder	4	1	–	42	2	2	8	–	–	7	–	6	–	–	–	–
Sleep-related disorders	820	5	–	684	4	45	565	3	1	408	–	456	9	32	20	–
Sleep disorders^(3)^	105	–	–	152	2	15	216	1	1	115	–	105	2	8	6	–
Insomnia^(4)^	608	3	–	423	1	21	284	1	–	241	–	293	5	18	12	–
Nightmare	32	1	–	49	–	4	16	1	–	12	–	22	1	–	1	–
Abnormal dreams	25	–	–	38	1	4	13	–	–	18	–	14	–	3	–	–
Poor quality sleep	50	1	–	22	–	1	36	–	–	22	–	22	1	3	1	–
Suicidal thoughts and behaviours	95	3	–	422	25	30	73	1	–	80	–	176	27	22	1	–
Completed suicide	5	–	–	28	–	2	4	–	–	2	–	27	–	1	–	–
Depression suicidal	7	–	–	26	–	1	2	–	–	4	–	4	3	–	–	–
Suicidal ideation	65	3	–	325	23	26	47	1	–	72	–	98	23	19	–	–
Suicide attempt	18	–	–	43	2	1	20	–	–	2	–	47	1	2	1	–

Bolded rows are categories and these are not single AEs, with the numbers representing the total reports for the respective category. Eating disorder is the only report in its category so it is also bolded. (1)Depressive disorders include depressive mood; depression; depressive symptoms; major depression. (2)Hallucinations include hallucination; hallucination, auditory; hallucination, mixed; hallucination, synesthetic; hallucination, visual. (3)Sleep disorders include sleep disorder; sleep disorder due to a general medical condition; sleep disorder due to general medical condition, insomnia type; sleep disorder due to general medical condition, hypersomnia type. (4)Insomnia includes insomnia; initial insomnia; middle insomnia; terminal insomnia. (5)As of the search end dates, no data on the adverse effects of Tirzepatide has been submitted to the CVAROD, nor has any been submitted to the DEAN for Lixisenatide

Furthermore, among the AEs investigated, the most frequently reported in each database were explored further. In FAERS, depressive disorders (n = 1,956, 14%), insomnia (n = 1,861, 13.2%) and anxiety (n = 1,851, 13.2%) were the top reported AEs. In CVAROD, depressive disorders (n = 65, 19.5%), suicidal ideation (n = 45, 13.5%) and anxiety (n = 41, 12.3%) were the most common. Meanwhile, in DAEN, suicidal ideation (n = 50, 25.1%), depressive disorders (n = 30, 15.1%) and anxiety (n = 24, 12.1%) were the highest reported (Table 1). Most psychiatric AEs were recorded from patients taking exenatide (n = 4,777, 32.9%), followed by semaglutide (n = 3,490, 24.1%), dulaglutide (n = 2,667, 18.4%), liraglutide (n = 2,215, 15.3%), tirzepatide (n = 1,282, 8.8%) and lixisenatide (n = 79, 0.5%) (Table 1).

### Reporting odds ratio results

RORs and CIs were calculated for psychiatric AEs of interest across all databases. The significant results are reported in Table 2.

**Table 2:** Significant ROR and CI results from the FAERS database

Medication	Symptom	ROR	Lower CI	Upper CI
Exenatide	Disorientation	1.32	1.14	1.52
	Nervousness	3.75	3.48	4.05
	Stress	1.90	1.73	2.08
Semaglutide	Depressed mood	1.48	1.25	1.76
	Depression	1.35	1.22	1.48
	Depressive symptoms	6.24	4.49	8.69
	Nervousness	1.59	1.32	1.91
	Panic attack	1.46	1.16	1.82
	Stress	1.24	1.06	1.45
	Depression suicidal	4.35	2.95	6.40
	Suicidal ideation	2.58	2.31	2.88
Dulaglutide	Eating disorder	1.47	1.26	1.71
	Sleep disorder due to general medical condition, insomnia type	2.93	2.35	3.66
Tirzepatide	Eating disorder	1.58	1.14	2.20
	Panic attack	1.79	1.37	2.35
	Sleep disorder due to general medical condition, insomnia type	6.13	4.73	7.95

These results suggest a statistically significant association between each medication and various psychological symptoms, with some symptoms (e.g., depressive symptoms for semaglutide and sleep disorders for tirzepatide) showing particularly strong associations.

The findings from the US database were used to guide investigations into the Australian DAEN and Canadian CVAROD databases to determine if similar trends could be identified. Six significant correlations with the US FAERS data were found in the Australian DAEN database, which are shown in Table 3.

**Table 3:** Significant ROR and CI results from DAEN which were also significant in FAERS

Medication	Symptom	ROR	Lower CI	Upper CI
Semaglutide	Depressed mood	4.97	1.85	13.38
	Depression	6.75	3.59	12.69
	Panic attack	3.56	1.14	11.12
	Stress	4.15	1.03	16.78
Dulaglutide	Eating disorder	17.66	2.45	127.37
	Sleep disorder due to general medical condition, insomnia type	259.13	31.07	2161.43

From the Canadian data, only one correlation with the US data was found. That was for semaglutide and suicidal ideation (ROR = 4.38 CI 2.97–6.47). Additionally, both the Australian and Canadian databases highlighted a strong link between liraglutide and depression (DAEN ROR = 2.51 CI 1.12–5.61; CVAROD ROR = 1.68 CI 1.12–2.51), a finding which was not reflected in the US data. A combined view of the significant findings sorted by symptoms can be seen in Table 4.

**Table 4:** Summary table of significant ROR and CI results across the FAERS database and corresponding significant results from DAEN and CVAROD

Symptom	Medication	ROR	Lower CI	Upper CI	Database
Depressed mood	Semaglutide	1.48	1.25	1.76	FAERS
	Semaglutide	4.97	1.85	13.38	DAEN
Depression	Semaglutide	1.35	1.22	1.48	FAERS
	Semaglutide	6.75	3.59	12.69	DAEN
	Liraglutide	2.51	1.12	5.61	DAEN
	Liraglutide	1.68	1.12	2.51	CVAROD
Panic attack	Semaglutide	1.46	1.16	1.82	FAERS
	Semaglutide	3.56	1.14	11.12	DAEN
Stress	Semaglutide	1.24	1.06	1.45	FAERS
	Semaglutide	4.15	1.03	16.78	DAEN
Suicidal ideation	Semaglutide	2.58	2.31	2.88	FAERS
	Semaglutide	4.38	2.97	6.47	CVAROD
Eating disorder	Dulaglutide	1.47	1.26	1.71	FAERS
	Dulaglutide	17.66	2.45	127.37	DAEN
Sleep disorder due to general medical condition, insomnia type	Dulaglutide	2.93	2.35	3.66	FAERS
	Dulaglutide	259.13	31.07	2161.43	DAEN

## Discussion

GLP-1 analogues have become increasingly popular in recent decades, primarily for their effectiveness in improving glycaemic control, promoting weight loss, and providing cardiovascular benefits [1–3]. While numerous studies have investigated the potential psychiatric AEs associated with GLP-1 analogues, their findings remain contradictory [8–13]. Our study was the first pharmacovigilance investigation that incorporated data from three different global databases to examine the potential psychiatric risks associated with GLP-1RAs. Using the relevant search dates (Supplementary Table 1), we identified a total of 16,678 psychiatric AE reports across the three databases for the GLP-1 analogues of interest (Supplementary Table 2). Notably, the number of psychiatric AE reports from FAERS alone (16,090) has doubled when compared to a previous pharmacovigilance study (8,240) which examined psychiatric AEs for all GLP-1 analogues in FAERS from Q1 2004—Q1 2023 [11], highlighting a significant increase in psychiatric AEs associated with GLP-1 analogues in the last 2 years.

More than 57% of reports occurred in patients aged 18 or older (42% unspecified) and about 64% were in females, highlighting the possible more frequent use of GLP-1 analogues among adult female populations. A previous study using the EudraVigilance database found similar results, showing that approximately 50% of reports came from the 18–64 year age group and 65% were female [19].

We observed several significant associations between certain GLP-1 analogues and specific psychiatric AEs. Treatment with semaglutide was highly associated with the occurrence of depressed mood, panic attack, and stress in both the FAERS and DAEN databases, and it was also notably linked to suicidal ideation in both the FAERS and CVAROD databases. Treatment with dulaglutide was highly associated with the occurrence of eating disorders and sleep disorders due to general medical condition, insomnia type in both the FAERS and DAEN databases while treatment with liraglutide demonstrated a strong relationship with depression in both the DAEN and CVAROD databases.

These findings were consistent with those of Chen et al. [11], who also reported significant associations between the overall class effect of GLP-1 analogues and symptoms such as stress, eating, and sleep disorder due to general medical condition-insomnia type. However, no positive associations were reported in their study between depression, depressed mood, panic attack, and suicidal ideation and GLP-1 analogues. Notably, a large cohort study by Kornelius et al. [23] found that GLP-1 analogues, particularly liraglutide and semaglutide, are associated with a 195% increased risk of depression and a 106% higher risk for suicidal ideation. Their findings, along with those of the present study, were also aligned with two other pharmacovigilance studies, which identified potential associations between GLP-1 analogues, particularly semaglutide, and an increased risk of suicidal thoughts and behaviors due to higher reporting probabilities [12, 13]. Conversely, a retrospective cohort study [10] reported a lower risk of incidence and recurrence of suicidal ideation in patients prescribed with semaglutide. Furthermore, a clinical trial involving 3,377 participants and a cohort study with 124,517 participants found that GLP-1 analogues were not associated with either suicidal ideation, depression, nor suicidal death [24, 25]. The observed differences across different studies suggest uncertainty regarding the psychiatric effects of GLP-1 analogues, indicating a need for further investigation.

The prevalence of psychiatric AEs with GLP-1 analogue use may be due to pre-existing psychiatric disorders in patients [11]. The databases used in this study did not contain information on any pre-existing psychiatric conditions in patients reporting AEs. While the association between GLP-1 analogue use and psychiatric AEs is still not clear, given the potentially serious consequences of psychiatric AEs, additional attention in drug selection and closer monitoring of AEs in patients is warranted. As suggested in other studies, further pharmacovigilance and clinical studies are required to establish the causality link and identify the mechanism between psychiatric AEs and the use of GLP-1 analogues [10–13, 25].

### Strengths

The use of three different databases based in three different countries provided a larger dataset than other studies to date. This increases statistical power allowing for more reliable identification of AEs that might not be apparent in country-specific studies with smaller samples. The countries included in this study have diverse populations which may influence responses to the drugs. This could offer insights into whether AE trends are consistent across different demographics, potentially highlighting safety concerns.

Additionally, the use of ROR to indicate the degree of association between drugs and AEs allows for a more straightforward comparison between each database and drug. Investigating a range of medications in a class allows for the identification of any potential differences in GLP-1 analogues and their AE profiles. This may aid clinicians in tailoring prescribing decisions in patients with pre-existing psychiatric conditions.

### Limitations

This study had limitations that must be considered when interpreting the results. The study relied on self-reported AEs from patients and prescribers, and this may be subject to bias leading to over- or under-reporting. Moreover, these reports were based on the reporter’s opinion, making it difficult to determine the specific cause of the event.

Another limitation was the lack of patient characteristics such as medical history, comorbidities, family history and lifestyle factors. This made it difficult to distinguish between a medicine-induced reaction and an event related to a patient’s ongoing health issues. Hence, it is difficult to determine a causal relationship between medication use and AEs. Note there are currently no clinically significant drug-drug interactions with any GLP-1 analogues therefore this was not assessed [26].

The total number of patients prescribed each medication in each country was unknown, so it was not possible to determine the true incidence of the AEs. In this case the ROR is the best statistical tool available to the investigators. The newer drugs have fewer reports since they have been on the market for a shorter period which made it difficult to compare these medicines to others that had been available for longer.

Hence, whilst these reports provide signals for potential drug risks, they should be interpreted with caution and be supplemented with clinical studies to confirm associations.

## Conclusion

Our study is the first pharmacovigilance study that involved multiple databases from the US, Canada and Australia in investigating the psychiatric AEs associated with GLP-1 analogues. Several significant associations between certain GLP-1 analogues and specific psychiatric AEs were observed. Although several limitations exist, this preliminary study provides valuable insights for further research into the psychiatric AEs associated with GLP-1 analogues and highlights the significance of ongoing monitoring and evaluations of these medications, especially considering the potentially serious consequences of psychiatric AEs. Healthcare professionals are encouraged to approach prescribing of these medications with caution, after weighing their potential risks and benefits.

## Supplementary Information

Below is the link to the electronic supplementary material.

Supplementary file1 (DOCX 163 KB)
