# Supplementary material for: Psychiatric adverse events linked to glucagon-like peptide 1 analogues: a disproportionality analysis in American, Canadian and Australian adverse event databases
Source: Int J Clin Pharm. 2025 Jun 16;47(6):1739–47. doi: 10.1007/s11096-025-01943-x (PMC12630159; doi:10.1007/s11096-025-01943-x)
Supplement: Supplementary file 1 — Supplementary file1 (DOCX 163 KB) [file 11096_2025_1943_MOESM1_ESM.docx]

| **Medication name** | **US Launch Dates^(1)^** | **Database Search End Date** | **Australian Launch Dates^(2)^** | **Database Search End Date** | **Canadian Launch Dates^(3)^** | **Database Search End Date** |
| --- | --- | --- | --- | --- | --- | --- |
| Exenatide | 28/04/2005 | 30/06/2024 | 05/02/2013 | 10/07/2024 | 16/02/2016 - 28/02/2022 Cancelled Post-Market | 31/03/2024 |
| Liraglutide | 25/1/2010 | 30/06/2024 | 24/12/2015 | 10/07/2024 | 27/05/2015 | 31/03/2024 |
| Lixisenatide | 27/07/2016 | 30/06/2024 | 10/04/2013 | 10/07/2024 | 12/09/2017 | 31/03/2024 |
| Dulaglutide | 18/09/2014 | 30/06/2024 | 19/01/2015 | 10/07/2024 | 08/01/2016 | 31/03/2024 |
| Semaglutide | 05/12/2017 | 30/06/2024 | 30/10/2020 | 10/07/2024 | 22/02/2018 | 31/03/2024 |
| Tirzepatide | 13/05/2022 | 30/06/2024 | 22/12/2022 | 10/07/2024 | 24/11/2022 | 31/03/2024 |

**Supplementary Table 1.** Date range for the available data for the GLP-1 analogues in the respective databases. This table displays each GLP-1 analogue, its launch date in each country and the end date of the search with regards to each database. The superscripts indicate the source for each launch date. (1) Food and Drug Authority, Drug Approvals and Databases. (2) Therapeutic Goods Administration (TGA). (3) Government of Canada, Drug Product Database.

**Supplementary Table 2.** Comparative table of all psychiatric AEs listed for each GLP-1 analogue in the three databases. This table displays the number of reports for all psychiatric AEs and categories from each database. Note that all bolded lines are categories and not single AEs, with the numbers representing the total reports for the respective category. As of the research end dates, no data on the adverse effects of Tirzepatide has been submitted to the CVAROD, nor has any been submitted to the DEAN for Lixisenatide.

|  | **Exenatide** | | | **Semaglutide** | | | **Dulaglutide** | | | **Tirzepatide** | | **Liraglutide** | | | **Lixisenatide** | | **Totals** |
| --- | --- | --- | --- | --- | --- | --- | --- | --- | --- | --- | --- | --- | --- | --- | --- | --- | --- |
| **Adverse event** | FAERS | DAEN | CVAROD | FAERS | DAEN | CVAROD | FAERS | DAEN | CVAROD | FAERS | DAEN | FAERS | DAEN | CVAROD | FAERS | CVAROD | ALL |
| **Aggression and Social Avoidance** | **72** | **1** | **-** | **66** | **-** | **4** | **35** | **1** | **-** | **18** | **-** | **47** | **-** | **3** | **-** | **-** | **247** |
| Aggression | 16 | - | - | 39 | - | 1 | 12 | - | - | 5 | - | 18 | - | - | - | - |  |
| Anger | 48 | 1 | - | 22 | - | 3 | 22 | 1 | - | 12 | - | 21 | - | 1 | - | - |  |
| Asocial Behaviour | - | - | - | 1 | - | - | - | - | - | - | - | - | - | - | - | - |  |
| Belligerence | - | - | - | - | - | - | - | - | - | - | - | 1 | - | - | - | - |  |
| Homicidal Ideation | 1 | - | - | 1 | - | - | - | - | - | 1 | - | 2 | - | - | - | - |  |
| Social Avoidant Behaviour | 6 | - | - | 2 | - | - | - | - | - | - | - | 3 | - | 2 | - | - |  |
| Violence-Related Symptom | 1 | - | - | 1 | - | - | 1 | - | - | - | - | 2 | - | - | - | - |  |
| **Agitation and Restlessness** | **346** | **-** | **-** | **129** | **3** | **8** | **130** | **1** | **-** | **58** | **1** | **120** | **2** | **5** | **6** | **-** | **809** |
| Agitation | 90 | - | - | 28 | 1 | 1 | 33 | 1 | - | 15 | - | 33 | 2 | 1 | 2 | - |  |
| Irritability | 217 | - | - | 78 | 2 | 7 | 82 | - | - | 32 | - | 61 | - | 3 | 3 | - |  |
| Restlessness | 39 | - | - | 23 | - | - | 15 | - | - | 11 | 1 | 26 | - | 1 | 1 | - |  |
| **Behavioural Disorders** | **58** | **-** | **-** | **52** | **3** | **4** | **33** | **-** | **1** | **22** | **-** | **43** | **-** | **1** | **4** | **-** | **221** |
| Abnormal Behaviour | 26 | - | - | 19 | 1 | 2 | 16 | - | 1 | 6 | - | 9 | - | - | 1 | - |  |
| Affect Lability | 4 | - | - | 3 | - | - | 6 | - | - | 1 | - | 10 | - | - | 1 | - |  |
| Affective Disorder | 7 | - | - | 9 | - | 2 | 3 | - | - | 4 | - | 8 | - | - | - | - |  |
| Alcohol Abuse | 2 | - | - | - | - | - | 1 | - | - | 1 | - | 1 | - | - | - | - |  |
| Alcoholic Hangover | - | - | - | - | - | - | 1 | - | - | - | - | - | - | - | - | - |  |
| Alcohol Use Disorder | - | - | - | 1 | - | - | - | - | - | 1 | - | - | - | - | - | - |  |
| Alcoholism | 1 | - | - | 1 | - | - | - | - | - | 1 | - | 1 | - | - | - | - |  |
| Behaviour Disorder | 3 | - | - | 3 | 1 | - | - | - | - | - | - | - | - | - | - | - |  |
| Binge Drinking | - | - | - | - | - | - | 1 | - | - | - | - | - | - | - | - | - |  |
| Borderline Personality Disorder | 1 | - | - | - | - | - | - | - | - | 1 | - | - | - | - | - | - |  |
| Disturbance In Social Behaviour | - | - | - | - | 1 | - | - | - | - | 1 | - | 1 | - | - | - | - |  |
| Drug Abuse | 6 | - | - | 9 | - | - | - | - | - | 1 | - | 2 | - | 1 | 1 | - |  |
| Drug Dependence | 5 | - | - | 1 | - | - | 2 | - | - | 4 | - | 7 | - | - | 1 | - |  |
| Histrionic Personality Disorder | 1 | - | - | - | - | - | - | - | - | - | - | - | - | - | - | - |  |
| Inappropriate Affect | 1 | - | - | - | - | - | 2 | - | - | - | - | 2 | - | - | - | - |  |
| Nicotine Dependence | - | - | - | 2 | - | - | 1 | - | - | 1 | - | - | - | - | - | - |  |
| Self-Destructive Behaviour | 1 | - | - | 3 | - | - | - | - | - | - | - | 2 | - | - | - | - |  |
| Sexually Inappropriate Behaviour | - | - | - | 1 | - | - | - | - | - | - | - | - | - | - | - | - |  |
| **Bipolar and Related Disorders** | **134** | **2** | **-** | **92** | **2** | **6** | **81** | **2** | **-** | **38** | **-** | **82** | **1** | **7** | **-** | **-** | **447** |
| Bipolar Disorder | 14 | - | - | 13 | - | - | 14 | - | - | 7 | - | 7 | - | - | - | - |  |
| Bipolar I Disorder | 2 | - | - | - | - | - | 2 | 1 | - | 1 | - | 1 | - | - | - | - |  |
| Euphoric Mood | 25 | - | - | 10 | - | - | 10 | - | - | 3 | - | 6 | - | 1 | - | - |  |
| Hypomania | 2 | - | - | 1 | - | - | 1 | - | - | 1 | - | - | - | - | - | - |  |
| Mania | 8 | 2 | - | 7 | - | - | 1 | - | - | 2 | - | 6 | - | - | - | - |  |
| Mood Altered | 44 | - | - | 35 | 1 | 5 | 29 | - | - | 16 | - | 26 | 1 | 4 | - | - |  |
| Mood Swings | 39 | - | - | 26 | 1 | 1 | 24 | 1 | - | 8 | - | 36 | - | 2 | - | - |  |
| **Cognitive and Emotional Intrusions** | **-** | **-** | **-** | **17** | **2** | **1** | **2** | **-** | **-** | **3** | **-** | **5** | **1** | **1** | **-** | **-** | **32** |
| Intrusive Thoughts | - | - | - | 8 | 1 | 1 | - | - | - | 3 | - | 1 | 1 | 1 | - | - |  |
| Morbid Thoughts | - | - | - | 2 | - | - | - | - | - | - | - | 1 | - | - | - | - |  |
| Negative Thoughts | - | - | - | 7 | 1 | - | 2 | - | - | - | - | 3 | - | - | - | - |  |
| **Communication Disorders** | **44** | **-** | **-** | **11** | **-** | **-** | **26** | **-** | **-** | **7** | **-** | **11** | **-** | **-** | **2** | **-** | **101** |
| Communication Disorder | 8 | - | - | 1 | - | - | 4 | - | - | 1 | - | 1 | - | - | 1 | - |  |
| Disorganised Speech | - | - | - | 1 | - | - | 1 | - | - | - | - | - | - | - | - | - |  |
| Dysphemia | 6 | - | - | 2 | - | - | 7 | - | - | 2 | - | 1 | - | - | - | - |  |
| Lack Of Spontaneous Speech | 1 | - | - | - | - | - | - | - | - | - | - | - | - | - | - | - |  |
| Logorrhoea | 5 | - | - | 1 | - | - | - | - | - | - | - | 3 | - | - | - | - |  |
| Mutism | - | - | - | - | - | - | - | - | - | - | - | 1 | - | - | - | - |  |
| Speech Disorder | 24 | - | - | 6 | - | - | 12 | - | - | 3 | - | 5 | - | - | 1 | - |  |
| Thought Blocking | - | - | - | - | - | - | 2 | - | - | 1 | - | - | - | - | - | - |  |
| **Confusion and Cognitive Changes** | **763** | **3** | **-** | **257** | **9** | **11** | **392** | **5** | **-** | **96** | **-** | **217** | **5** | **7** | **9** | **-** | **1774** |
| Amnesia | 25 | - | - | 11 | - | - | 19 | - | - | 7 | - | 8 | - | - | - | - |  |
| Bradyphrenia | 4 | - | - | 1 | - | - | 9 | - | - | 1 | - | 2 | - | - | - | - |  |
| Confusional State | 424 | 2 | - | 141 | 4 | 6 | 194 | 3 | - | 37 | - | 115 | - | 4 | 6 | - |  |
| Deja Vu | 2 | - | - | - | - | - | - | - | - | - | - | 1 | - | - | - | - |  |
| Derailment | 1 | - | - | - | - | - | - | - | - | - | - | - | - | - | - | - |  |
| Disorientation | 183 | - | - | 33 | - | 1 | 34 | - | - | 14 | - | 32 | - | 2 | - | - |  |
| Impaired Reasoning | - | - | - | - | - | - | 1 | - | - | - | - | - | - | - | - | - |  |
| Mental Disorder | 45 | 1 | - | 41 | 5 | 2 | 71 | 2 | - | 19 | - | 25 | 4 | 1 | 2 | - |  |
| Mental Disorder Due To A General Medical Condition | 3 | - | - | - | - | - | - | - | - | - | - | - | - | - | - | - |  |
| Mental Fatigue | 1 | - | - | 2 | - | - | 4 | - | - | 4 | - | 2 | - | - | - | - |  |
| Mental Status Changes | 25 | - | - | 10 | - | 1 | 4 | - | - | 5 | - | 12 | - | - | - | - |  |
| Thinking Abnormal | 48 | - | - | 16 | - | 1 | 53 | - | - | 9 | - | 18 | 1 | - | 1 | - |  |
| Time Perception Altered | 2 | - | - | 2 | - | - | 3 | - | - | - | - | 2 | - | - | - | - |  |
| **Depressive Disorders** | **524** | **4** | **-** | **616** | **14** | **34** | **291** | **2** | **-** | **174** | **-** | **356** | **10** | **31** | **9** | **-** | **2065** |
| Adjustment Disorder With Depressed Mood | - | - | - | 3 | - | - | - | - | - | - | - | 1 | - | - | - | - |  |
| Depressed Mood* | 130 | - | - | 131 | 4 | 11 | 83 | - | - | 42 | - | 59 | 3 | 5 | 4 | - |  |
| Depression* | 378 | 4 | - | 430 | 10 | 21 | 202 | 2 | - | 131 | - | 286 | 6 | 24 | 5 | - |  |
| Depressive Symptom* | 2 | - | - | 36 | - | 2 | 2 | - | - | - | - | 5 | - | 2 | - | - |  |
| Grief Reaction | 1 | - | - | 1 | - | - | - | - | - | - | - | - | - | - | - | - |  |
| Major Depression* | 10 | - | - | 12 | - | - | 3 | - | - | 1 | - | 4 | 1 | - | - | - |  |
| Persistent Depressive Disorder | 1 | - | - | 1 | - | - | - | - | - | - | - | - | - | - | - | - |  |
| Perinatal Depression | - | - | - | 1 | - | - | - | - | - | - | - | 1 | - | - | - | - |  |
| Seasonal Affective Disorder | 2 | - | - | 1 | - | - | 1 | - | - | - | - | - | - | - | - | - |  |
| **Dissociative Disorders** | **34** | **-** | **-** | **27** | **2** | **2** | **16** | **1** | **-** | **7** | **-** | **23** | **-** | **1** | **1** | - | **114** |
| Daydreaming | 4 | - | - | 2 | - | - | 5 | - | - | - | - | 3 | - | - | - | - |  |
| Delirium | 18 | - | - | 12 | 1 | 1 | 10 | 1 | - | 4 | - | 15 | - | 1 | 1 | - |  |
| Delirium Tremens | - | - | - | - | - | - | - | - | - | - | - | 1 | - | - | - | - |  |
| Depersonalisation/Derealisation Disorder | 1 | - | - | 1 | - | - | - | - | - | 1 | - | - | - | - | - | - |  |
| Derealisation | 2 | - | - | 2 | - | - | 1 | - | - | 2 | - | 1 | - | - | - | - |  |
| Dissociation | 8 | - | - | 10 | - | 1 | - | - | - | - | - | 3 | - | - | - | - |  |
| Dissociative Amnesia | 1 | - | - | - | 1 | - | - | - | - | - | - | - | - | - | - | - |  |
| **Eating Disorders** | **121** | **1** | **-** | **116** | **1** | **5** | **184** | **1** | **-** | **48** | **-** | **54** | **1** | **3** | **3** | **-** | **538** |
| Anorexia Nervosa | - | - | - | 1 | - | - | - | - | - | 2 | - | - | - | - | - | - |  |
| Appetite Disorder | 20 | - | - | 47 | - | - | 4 | - | - | - | - | 6 | - | - | - | - |  |
| Binge Eating | 9 | - | - | 14 | - | - | 4 | - | - | 6 | - | 5 | - | - | 1 | - |  |
| Bulimia Nervosa | - | - | - | 3 | 1 | - | - | - | - | - | - | 5 | 1 | - | - | - |  |
| Eating Disorder | 83 | 1 | - | 44 | - | 5 | 172 | 1 | - | 36 | - | 33 | - | 2 | 2 | - |  |
| Fear Of Eating | - | - | - | 2 | - | - | 4 | - | - | 3 | - | 2 | - | - | - | - |  |
| Merycism | 2 | - | - | 1 | - | - | - | - | - | - | - | - | - | - | - | - |  |
| Pica | 1 | - | - | - | - | - | - | - | - | - | - | - | - | - | - | - |  |
| Selective Eating Disorder | - | - | - | 3 | - | - | - | - | - | 1 | - | 2 | - | - | - | - |  |
| Self-Induced Vomiting | 5 | - | - | 1 | - | - | - | - | - | - | - | 1 | - | 1 | - | - |  |
| Vomiting Psychogenic | 1 | - | - | - | - | - | - | - | - | - | - | - | - | - | - | - |  |
| **Emotional Disorders and Symptoms** | **155** | **-** | **-** | **95** | **6** | **5** | **60** | **1** | **-** | **22** | **-** | **50** | **2** | **9** | **5** | **-** | **410** |
| Abulia | 2 | - | - | 1 | - | - | - | - | - | - | - | - | - | - | - | - |  |
| Anhedonia | 3 | - | - | 3 | - | - | - | - | - | 1 | - | 1 | - | - | - | - |  |
| Apathy | 13 | - | - | 26 | 1 | 2 | 12 | - | - | 5 | - | 14 | 1 | 3 | - | - |  |
| Aversion | 1 | - | - | 4 | - | - | - | - | - | 1 | - | 1 | - | - | - | - |  |
| Decreased Interest | 4 | - | - | 5 | - | 1 | 4 | - | - | 2 | - | 3 | - | 4 | - | - |  |
| Discouragement | - | - | - | 12 | - | - | - | - | - | - | - | 4 | - | - | - | - |  |
| Dysphoria | 2 | - | - | - | 1 | - | 1 | - | - | - | - | 2 | 1 | - | - | - |  |
| Emotional Disorder | 23 | - | - | 18 | 2 | - | 17 | 1 | - | 6 | - | 13 | - | 1 | - | - |  |
| Emotional Poverty | - | - | - | 2 | - | - | - | - | - | 4 | - | 1 | - | - | - | - |  |
| Feeling Of Despair | 6 | - | - | 8 | - | - | 5 | - | - | - | - | 3 | - | - | - | - |  |
| Feelings Of Worthlessness | 1 | - | - | 1 | - | - | - | - | - | 1 | - | - | - | - | - | - |  |
| Frustration Tolerance Decreased | 94 | - | - | 11 | - | 1 | 18 | - | - | 2 | - | 6 | - | - | 5 | - |  |
| Indifference | 1 | - | - | - | 1 | - | - | - | - | - | - | - | - | - | - | - |  |
| Self Esteem Decreased | - | - | - | 3 | - | 1 | - | - | - | - | - | 1 | - | - | - | - |  |
| Tearfulness | 5 | - | - | 1 | 1 | - | 3 | - | - | - | - | 1 | - | 1 | - | - |  |
| **Functional Neurologic Disorders** | **10** | **-** | **-** | **10** | **1** | **-** | **3** | **-** | **-** | **1** | **-** | **-** | **1** | **-** | **-** | **-** | **26** |
| Automatism | 1 | - | - | 1 | - | - | - | - | - | - | - | - | - | - | - | - |  |
| Conversion Disorder | 3 | - | - | 1 | 1 | - | 1 | - | - | 1 | - | - | - | - | - | - |  |
| Dystonia | - | - | - | 1 | - | - | 1 | - | - | - | - | - | - | - | - | - |  |
| Head Banging | 1 | - | - | - | - | - | 1 | - | - | - | - | - | - | - | - | - |  |
| Tic | 5 | - | - | 7 | - | - | - | - | - | - | - | - | 1 | - | - | - |  |
| **Generalised Anxiety** | **1283** | **1** | **1** | **557** | **11** | **22** | **500** | **3** | **-** | **253** | **1** | **393** | **9** | **23** | **18** | **-** | **3075** |
| Anxiety | 574 | 1 | 1 | 434 | 11 | 19 | 327 | 2 | - | 217 | 1 | 291 | 9 | 21 | 8 | - |  |
| Anxiety Disorder | 8 | - | - | 6 | - | - | 2 | - | - | - | - | 8 | - | - | - | - |  |
| Bruxism | 1 | - | - | 1 | - | - | - | - | - | 1 | - | 2 | - | - | - | - |  |
| Generalised Anxiety Disorder | 1 | - | - | - | - | - | 2 | - | - | - | - | - | - | - | - | - |  |
| Hypervigilance | 1 | - | - | - | - | - | - | - | - | - | - | - | - | - | - | - |  |
| Illness Anxiety Disorder | - | - | - | 2 | - | - | 4 | - | - | - | - | 1 | - | - | - | - |  |
| Mixed Anxiety And Depressive Disorder | 1 | - | - | 1 | - | - | 1 | - | - | - | - | - | - | - | - | - |  |
| Nervousness | 695 | - | - | 113 | - | 3 | 163 | 1 | - | 33 | - | 90 | - | 2 | 10 | - |  |
| Neurosis | 2 | - | - | - | - | - | - | - | - | 1 | - | - | - | - | - | - |  |
| Onychophagia | - | - | - | - | - | - | 1 | - | - | - | - | 1 | - | - | - | - |  |
| Social Anxiety Disorder | - | - | - | - | - | - | - | - | - | 1 | - | - | - | - | - | - |  |
| **Hallucinations** | **53** | **2** | **-** | **54** | **2** | **5** | **35** | **-** | **-** | **11** | **-** | **19** | **1** | **2** | **1** | - | **185** |
| Autoscopy | - | - | - | 3 | 1 | - | 1 | - | - | 1 | - | - | - | - | - | - |  |
| Hallucination | 41 | 1 | - | 32 | - | 3 | 30 | - | - | 8 | - | 15 | 1 | 2 | - | - |  |
| Hallucination, Auditory* | 2 | - | - | 14 | 1 | 2 | - | - | - | 2 | - | 1 | - | - | - | - |  |
| Hallucinations, Mixed* | 2 | - | - | - | - | - | - | - | - | - | - | - | - | - | - | - |  |
| Hallucination, Synaesthetic* | - | - | - | - | - | - | 1 | - | - | - | - | - | - | - | - | - |  |
| Hallucination, Visual* | 8 | 1 | - | 5 | - | - | 3 | - | - | - | - | 3 | - | - | 1 | - |  |
| **Impulse-Control Disorders** | **10** | **-** | **-** | **9** | **1** | **-** | **4** | **-** | **-** | **8** | **-** | **9** | **1** | **-** | **-** | - | **42** |
| Compulsions | - | - | - | - | - | - | - | - | - | - | - | 2 | 1 | - | - | - |  |
| Dermatillomania | - | - | - | 1 | - | - | - | - | - | - | - | - | - | - | - | - |  |
| Impulse-Control Disorder | - | - | - | - | 1 | - | - | - | - | - | - | 6 | - | - | - | - |  |
| Obsessive Thoughts | 5 | - | - | 4 | - | - | - | - | - | - | - | - | - | - | - | - |  |
| Obsessive-Compulsive Disorder | 5 | - | - | 4 | - | - | 4 | - | - | 8 | - | 1 | - | - | - | - |  |
| **Learning Disorders** | **9** | **-** | **-** | **5** | **-** | **-** | **7** | **-** | **-** | **2** | **-** | **1** | **-** | **-** | **-** | **-** | **24** |
| Learning Disability | **-** | **-** | **-** | 1 | - | - | 3 | - | - | - | - | - | - | - | - | - |  |
| Learning Disorder | 3 | - | - | 1 | - | - | 4 | - | - | - | - | - | - | - | - | - |  |
| Reading Disorder | 6 | - | - | 3 | - | - | - | - | - | 2 | - | 1 | - | - | - | - |  |
| **Misc** | **56** | **-** | **-** | **33** | **-** | **-** | **27** | **-** | **-** | **4** | **-** | **16** | **1** | **-** | **-** | - | **137** |
| Catatonia | 1 | - | - | 2 | - | - | 1 | - | - | - | - | 1 | - | - | - | - |  |
| Encopresis | 1 | - | - | - | - | - | - | - | - | - | - | - | - | - | - | - |  |
| Enuresis | 1 | - | - | 5 | - | - | 3 | - | - | - | - | - | - | - | - | - |  |
| Laziness | 4 | - | - | 5 | - | - | 10 | - | - | 2 | - | 2 | - | - | - | - |  |
| Listless | 28 | - | - | 2 | - | - | 6 | - | - | 1 | - | 5 | - | - | - | - |  |
| Near Death Experience | 14 | - | - | 16 | - | - | 1 | - | - | 1 | - | 6 | - | - | - | - |  |
| Psychiatric Decompensation | 1 | - | - | - | - | - | 1 | - | - | - | - | - | - | - | - | - |  |
| Psychiatric Symptom | 3 | - | - | 2 | - | - | 3 | - | - | - | - | 1 | 1 | - | - | - |  |
| Psychological Trauma | 1 | - | - | - | - | - | - | - | - | - | - | - | - | - | - | - |  |
| Somatic Symptom Disorder | 2 | - | - | 1 | - | - | 2 | - | - | - | - | 1 | - | - | - | - |  |
| **Neurodevelopmental disorders** | **11** | **-** | **-** | **12** | **-** | **1** | **10** | **-** | **-** | **1** | **-** | **2** | **-** | **-** | **1** | **-** | **38** |
| Attention Deficit Hyperactivity Disorder | 10 | - | - | 8 | - | 1 | 5 | - | - | 1 | - | 2 | - | - | - | - |  |
| Autism Spectrum Disorder | 1 | - | - | 3 | - | - | 2 | - | - | - | - | - | - | - | - | - |  |
| Change In Sustained Attention | - | - | - | 1 | - | - | - | - | - | - | - | - | - | - | - | - |  |
| Distractibility | - | - | - | - | - | - | 3 | - | - | - | - | - | - | - | 1 | - |  |
| **Panic and Stress Responses** | **653** | **1** | **2** | **292** | **7** | **10** | **384** | **1** | **-** | **124** | **-** | **133** | **4** | **5** | **9** | **1** | **1626** |
| Emotional Distress | 41 | - | - | 27 | 1 | - | 13 | - | - | 9 | - | 10 | - | 2 | 1 | 1 |  |
| Flashback | - | - | - | - | - | - | - | - | - | - | - | - | 1 | - | - | - |  |
| Panic Attack | 54 | - | - | 77 | 3 | 2 | 37 | - | - | 53 | - | 33 | 2 | 1 | 1 | - |  |
| Panic Disorder | 16 | - | - | 4 | - | - | 13 | - | - | 5 | - | 3 | - | - | - | - |  |
| Panic Reaction | 38 | - | - | 15 | - | - | 13 | - | - | 5 | - | 6 | - | - | 1 | - |  |
| Postpartum Stress Disorder | - | - | - | - | - | - | 1 | - | - | - | - | - | - | - | - | - |  |
| Post-Traumatic Stress Disorder | 8 | - | - | 6 | 1 | - | 17 | 1 | - | 2 | - | 8 | 1 | - | - | - |  |
| Stress | 462 | 1 | 1 | 157 | 2 | 8 | 282 | - | - | 45 | - | 70 | - | 2 | 6 | - |  |
| Stressed Eating | - | - | - | - | - | - | - | - | - | 1 | - | - | - | - | - | - |  |
| Tachyphrenia | 3 | - | 1 | 4 | - | - | 1 | - | - | 4 | - | 1 | - | - | - | - |  |
| Tension | 31 | - | - | 2 | - | - | 7 | - | - | - | - | 2 | - | - | - | - |  |
| **Phobias** | **126** | **-** | **-** | **65** | **-** | **2** | **86** | **1** | **-** | **22** | **-** | **18** | **-** | **-** | **4** | **-** | **324** |
| Agoraphobia | - | - | - | 2 | - | - | - | - | - | - | - | 1 | - | - | **-** | - |  |
| Claustrophobia | 4 | - | - | - | - | - | 2 | - | - | - | - | 1 | - | - | 1 | - |  |
| Fear | 76 | - | - | 26 | - | 2 | 39 | 1 | - | 9 | - | 10 | - | - | 2 | - |  |
| Fear Of Death | 3 | - | - | 3 | - | - | 2 | - | - | - | - | 1 | - | - | - | - |  |
| Fear Of Disease | - | - | - | - | - | - | 1 | - | - | - | - | 1 | - | - | - | - |  |
| Fear Of Falling | 2 | - | - | 1 | - | - | 1 | - | - | - | - | 3 | - | - | - | - |  |
| Fear Of Injection | 38 | - | - | 30 | - | - | 39 | - | - | 13 | - | - | - | - | 1 | - |  |
| Fear Of Open Spaces | - | - | - | 1 | - | - | - | - | - | - | - | - | - | - | - | - |  |
| Fear Of Weight Gain | - | - | - | 1 | - | - | - | - | - | - | - | - | - | - | - | - |  |
| Fear-Related Avoidance Of Activities | - | - | - | 1 | - | - | - | - | - | - | - | - | - | - | - | - |  |
| Frigophobia | - | - | - | - | - | - | 1 | - | - | - | - | - | - | - | - | - |  |
| Phobia | 3 | - | - | - | - | - | 1 | - | - | - | - | 1 | - | - | - | - |  |
| **Psychotic Disorders** | **29** | **1** | **-** | **67** | **5** | **2** | **33** | **1** | **-** | **11** | **-** | **22** | **1** | **-** | **-** | **-** | **172** |
| Acute Psychosis | 1 | - | - | - | - | - | - | - | - | 1 | - | 1 | - | - | - | - |  |
| Delusion | 3 | - | - | 4 | - | - | 6 | - | - | - | - | - | - | - | - | - |  |
| Delusion Of Grandeur | 1 | - | - | - | - | - | - | - | - | - | - | - | - | - | - | - |  |
| Paranoia | 11 | - | - | 9 | 2 | - | 11 | - | - | 3 | - | 5 | 1 | - | - | - |  |
| Paranoid Personality Disorder | - | - | - | 1 | - | - | 1 | - | - | - | - | - | - | - | - | - |  |
| Persecutory Delusion | 1 | - | - | - | - | - | - | - | - | - | - | 1 | - | - | - | - |  |
| Psychotic Behaviour | 1 | - | - | 1 | - | - | - | - | - | - | - | 2 | - | - | - | - |  |
| Psychotic Disorder | 4 | 1 | - | 42 | 2 | 2 | 8 | - | - | 7 | - | 6 | - | - | - | - |  |
| Psychotic Symptom | - | - | - | 1 | - | - | 1 | - | - | - | - | - | - | - | - | - |  |
| Schizoaffective Disorder | - | - | - | 1 | - | - | - | - | - | - | - | - | - | - | - | - |  |
| Schizoaffective Disorder Bipolar Type | - | - | - | 1 | - | - | - | - | - | - | - | 1 | - | - | - | - |  |
| Schizophrenia | 7 | - | - | 7 | 1 | - | 6 | 1 | - | - | - | 6 | - | - | - | - |  |
| **Sexual Dysfunction** | **46** | **-** | **-** | **36** | **1** | **1** | **11** | **-** | **-** | **16** | **-** | **23** | **-** | **-** | **-** | **-** | **134** |
| Anorgasmia | 2 | - | - | 2 | - | - | 1 | - | - | 2 | - | - | - | - | - | - |  |
| Disturbance In Sexual Arousal | 2 | - | - | - | - | - | - | - | - | 1 | - | - | - | - | - | - |  |
| Excessive Masturbation | - | - | - | 1 | - | - |  | - | - | - | - | - | - | - | - | - |  |
| Libido Decreased | 15 | - | - | 11 | - | - | - | - | - | 9 | - | 15 | - | - | - | - |  |
| Libido Disorder | 1 | - | - | 1 | - | - | 1 | - | - | - | - | - | - | - | - | - |  |
| Libido Increased | 12 | - | - | 2 | - | - | - | - | - | 1 | - | - | - | - | - | - |  |
| Loss Of Libido | 2 | - | - | 1 | 1 | 1 | 1 | - | - | - | - | 1 | - | - | - | - |  |
| Orgasm Abnormal | 1 | - | - | 1 | - | - | 1 | - | - | - | - | - | - | - | - | - |  |
| Personality Change | 9 | - | - | 15 | - | - | 7 | - | - | 3 | - | 4 | - | - | - | - |  |
| Premature Ejaculation | 1 | - | - | - | - | - | - | - | - | - | - | 3 | - | - | - | - |  |
| Psychogenic Erectile Dysfunction | 1 | - | - | 1 | - | - | - | - | - | - | - | - | - | - | - | - |  |
| Sexual Inhibition | - | - | - | 1 | - | - | - | - | - | - | - | - | - | - | - | - |  |
| **Sleep Disorders** | **825** | **5** | **-** | **701** | **4** | **49** | **571** | **3** | **1** | **415** | **-** | **477** | **9** | **32** | **22** | **-** | **3114** |
| Abnormal Dreams | 25 | - | - | 38 | 1 | 4 | 13 | - | - | 18 | - | 14 | - | 3 | **-** | - |  |
| Abnormal Sleep-Related Event | 1 | - | - | - | - | - | - | - | - | - | - | - | - | - | 1 | - |  |
| Initial Insomnia* | 11 | - | - | 12 | - | - | 6 | - | - | 6 | - | 9 | 1 | 2 | 1 | - |  |
| Insomnia | 574 | 3 | - | 387 | 1 | 16 | 267 | 1 | - | 227 | - | 282 | 4 | 16 | 11 | - |  |
| Loss Of Dreaming | - | - | - | 1 | - | - | - | - | - | - | - | - | - | - | - | - |  |
| Middle Insomnia* | 20 | - | - | 24 | - | 5 | 10 | - | - | 8 | - | 2 | - | - | - | - |  |
| Nightmare | 32 | 1 | - | 49 | - | 4 | 16 | 1 | - | 12 | - | 22 | 1 | - | 1 | - |  |
| Parasomnia | - | - | - | 1 | - | - | - | - | - | - | - | - | - | - | - | - |  |
| Poor Quality Sleep | 50 | 1 | - | 22 | - | 1 | 36 | - | - | 22 | - | 22 | 1 | 3 | 1 | - |  |
| Rapid Eye Movement Sleep Behaviour Disorder | - | - | - | 1 | - | 1 | - | - | - | - | - | - | - | - | - | - |  |
| Rapid Eye Movements Sleep Abnormal | - | - | - | - | - | - | - | - | - | 1 | - | - | - | - | - | - |  |
| Sleep Deficit | - | - | - | 2 | - | - | 3 | - | - | 3 | - | 1 | - | - | - | - |  |
| Sleep Disorder* | 90 | - | - | 75 | 2 | 11 | 66 | - | 1 | 27 | - | 35 | 1 | 3 | 6 | - |  |
| Sleep Disorder Due To A General Medical Condition. | 6 | - | - | 73 | - | 3 | 69 | - | - | 28 | - | 68 | - | 5 | - | - |  |
| Sleep Disorder Due To General Medical Condition, Hypersomnia type. | 2 | - | - | - | - | - | - | - | - | 1 | - | - | - | - | - | - |  |
| Sleep Disorder Due To General Medical Condition, Insomnia.type. | 7 | - | - | 4 | - | 1 | 81 | 1 | - | 59 | - | 2 | 1 | - | - | - |  |
| Sleep Talking | 2 | - | - | 3 | - | 1 | - | - | - | - | - | 1 | - | - | - | - |  |
| Sleep Terror | 1 | - | - | 4 | - | - | - | - | - | 1 | - | - | - | - | 1 | - |  |
| Sleep-Related Eating Disorder | - | - | - | 4 | - | 1 | 1 | - | - | - | - | - | - | - | - | - |  |
| Somnambulism | 1 | - | - | - | - | 1 | - | - | - | 2 | - | 18 | - | - | - | - |  |
| Sopor | - | - | - | - | - | - | - | - | - | - | - | 1 | - | - | - | - |  |
| Somniphobia | - | - | - | 1 | - | - | 2 | - | - | - | - | - | - | - | - | - |  |
| Terminal Insomnia | 3 | - | - | - | - | - | 1 | - | - | - | - | - | - | - | - | - |  |
| **Suicidal Thoughts and Behaviours** | **103** | **3** | **-** | **446** | **25** | **31** | **77** | **1** | **-** | **84** | **-** | **201** | **27** | **24** | **1** | **-** | **1023** |
| Completed Suicide | 5 | - | - | 28 | - | 2 | 4 | - | - | 2 | - | 27 | - | 1 | - | - |  |
| Depression Suicidal | 7 | - | - | 26 | - | 1 | 2 | - | - | 4 | - | 4 | 3 | - | - | - |  |
| Intentional Self-Injury | 4 | - | - | 8 | - | - | 2 | - | - | 1 | - | 4 | - | 2 | - | - |  |
| Self-Injurious Ideation | 2 | - | - | 9 | - | - | - | - | - | 2 | - | 10 | - | - | - | - |  |
| Suicidal Behaviour | 2 | - | - | 5 | - | 1 | 2 | - | - | 1 | - | 5 | - | - | - | - |  |
| Suicidal Ideation | 65 | 3 | - | 325 | 23 | 26 | 47 | 1 | - | 72 | - | 98 | 23 | 19 | - | - |  |
| Suicide Attempt | 18 | - | - | 43 | 2 | 1 | 20 | - | - | 2 | - | 47 | 1 | 2 | 1 | - |  |
| Suicide Threat | - | - | - | 2 | - | - | - | - | - | - | - | 1 | - | - | - | - |  |
| Suspected Suicide | - | - | - | - | - | - | - | - | - | - | - | 5 | - | - | - | - |  |
| **Grand Totals** | **5465** | **24** | **3** | **3765** | **99** | **203** | **2998** | **24** | **2** | **1445** | **2** | **2322** | **76** | **154** | **95** | **1** | **16678** |

**Supplementary Table 3.** Distribution of sex of the individuals experiencing the reported AEs across the three databases for the GLP-1 analogues of interest.

| Drug | Database | | Male | | Female | | Not Specified | | **Totals** | |
| --- | --- | --- | --- | --- | --- | --- | --- | --- | --- | --- |
|  |  | Count | | Percentage (%) | Count | Percentage (%) | Count | Percentage (%) | Count | Percentage  (%) |
| Exenatide | FAERS | 1347 | | 10.83 | 2935 | 23.60 | 62 | 0.50 | 4344 | 34.93 |
|  | DAEN | 9 | | 0.07 | 8 | 0.06 | - | - | 17 | 0.14 |
|  | CVAROD | - | | - | 2 | 0.02 | - | - | 2 | 0.02 |
| Dulaglutide | FAERS | 785 | | 6.31 | 1451 | 11.67 | 156 | 1.25 | 2392 | 19.23 |
|  | DAEN | 3 | | 0.02 | 12 | 0.10 | 1 | 0.01 | 16 | 0.13 |
|  | CVAROD | 1 | | 0.01 | 1 | 0.01 | - | - | 2 | 0.02 |
| Liraglutide | FAERS | 554 | | 4.45 | 1093 | 8.79 | 74 | 0.59 | 1721 | 13.84 |
|  | DAEN | 15 | | 0.12 | 29 | 0.23 | 7 | 0.06 | 51 | 0.41 |
|  | CVAROD | 10 | | 0.08 | 88 | 0.71 | 3 | 0.02 | 101 | 0.81 |
| Tirzepatide | FAERS | 245 | | 1.97 | 731 | 5.88 | 120 | 0.96 | 1096 | 8.81 |
|  | DEAN | 1 | | 0.01 | 1 | 0.01 | - | - | 2 | 0.02 |
|  | CVAROD | - | | - | - | - | - | - | - | - |
| Semaglutide | FAERS | 730 | | 5.87 | 1558 | 12.53 | 161 | 1.29 | 2449 | 19.69 |
|  | DAEN | 20 | | 0.16 | 29 | 0.23 | 3 | 0.02 | 52 | 0.42 |
|  | CVAROD | 40 | | 0.32 | 4 | 0.03 | 69 | 0.55 | 113 | 0.91 |
| Lixisenatide | FAERS | 24 | | 0.19 | 48 | 0.39 | 7 | 0.06 | 79 | 0.64 |
|  | DAEN | - | | - | - | - | - | - | - | - |
|  | CVAROD | 1 | | 0.01 | - | - | - | - | 1 | 0.01 |
| **Totals** | **ALL** | **3785** | | **30.43** | **7990** | **64.24** | **663** | **5.33** | **12438** | **100.00** |

**Supplementary Table 4.** Distribution of age ranges of the individuals experiencing the reported AEs across the three databases for the GLP-1 analogues of interest.

| Ages |  | Not Specified | | <18 |  | 18-44 |  | 45-65 |  | 65< |  | **Totals** |  |
| --- | --- | --- | --- | --- | --- | --- | --- | --- | --- | --- | --- | --- | --- |
|  |  | Count | Percentage (%) | Count | Percentage (%) | Count | Percentage  (%) | Count | Percentage  (%) | Count | Percentage  (%) | Count | Percentage (%) |
| Exenatide | FAERS | 1514 | 12.17 | 4 | 0.03 | 216 | 1.74 | 1728 | 13.89 | 882 | 7.09 | 4344 | 34.93 |
|  | DAEN | 2 | 0.02 | - | - | 2 | 0.02 | 9 | 0.07 | 4 | 0.03 | 17 | 0.14 |
|  | CVAROD | 1 | 0.01 | - | - | - | - | - | - | 1 | 0.01 | 2 | 0.02 |
| Dulaglutide | FAERS | 1504 | 12.09 | 2 | 0.02 | 89 | 0.72 | 432 | 3.47 | 365 | 2.93 | 2392 | 19.23 |
|  | DAEN | 6 | 0.05 | - | - | 1 | 0.01 | 6 | 0.05 | 3 | 0.02 | 16 | 0.13 |
|  | CVAROD | 1 | 0.01 | - | - | - | - | - | - | 1 | 0.01 | 2 | 0.02 |
| Liraglutide | FAERS | 603 | 4.85 | 10 | 0.08 | 188 | 1.51 | 620 | 4.98 | 300 | 2.41 | 1721 | 13.84 |
|  | DAEN | 31 | 0.25 | 1 | 0.01 | 6 | 0.05 | 12 | 0.10 | 1 | 0.01 | 51 | 0.41 |
|  | CVAROD | 30 | 0.24 | - | - | 20 | 0.16 | 38 | 0.31 | 13 | 0.10 | 101 | 0.81 |
| Tirzepatide | FAERS | 574 | 4.61 | - | - | 135 | 1.09 | 283 | 2.28 | 104 | 0.84 | 1096 | 8.81 |
|  | DAEN | - | - | - | - | 2 | 0.02 | - | - | - | - | 2 | 0.02 |
|  | CVAROD | - | - | - | - | 0 | 0.00 | - | - | - | - | - | - |
| Semaglutide | FAERS | 945 | 7.60 | 3 | 0.02 | 328 | 2.64 | 725 | 5.83 | 448 | 3.60 | 2449 | 19.69 |
|  | DEAN | 9 | 0.07 | - | - | 13 | 0.10 | 23 | 0.18 | 7 | 0.06 | 52 | 0.42 |
|  | CVAROD | 17 | 0.14 | - | - | 20 | 0.16 | 48 | 0.39 | 28 | 0.23 | 113 | 0.91 |
| Lixisenatide | FEARS | 29 | 0.23 | - | - | 8 | 0.06 | 21 | 0.17 | 21 | 0.17 | 79 | 0.64 |
|  | DEAN | - | - | - | - | - | - | - | - | - | - | - | - |
|  | CVAROD | - | - | - | - | - | - | - | - | 1 | 0.01 | 1 | 0.01 |
| **Totals** | **ALL** | **5266** | **42.34** | **20** | **0.16** | **1028** | **8.26** | **3945** | **31.72** | **2179** | **17.52** | **12438** | **100.00** |
